# Supporting data for analysis of the *Helicobacter pylori* exoproteome

**DOI:** 10.1016/j.dib.2015.10.008

**Published:** 2015-10-17

**Authors:** Christina A. Snider, Bradley J. Voss, W. Hayes McDonald, Timothy L. Cover

**Affiliations:** a Department of Medicine, Nashville, TN, USA; b Department of Pathology, Microbiology and Immunology, Nashville, TN, USA; c Proteomics Laboratory, Mass Spectrometry Research Center, Nashville, TN, USA; d Department of Biochemistry, Nashville, TN, USA; e Vanderbilt University School of Medicine; Veterans Affairs Tennessee Valley Healthcare System, Nashville, TN, USA

**Keywords:** Mass spectrometry, Proteomics, Bacterial protein secretion, Secretome, Exoproteome, Gastric cancer, Peptic ulcer disease

## Abstract

The goal of this research was to analyze the composition of the *Helicobacter pylori* exoproteome at multiple phases of bacterial growth (Snider et al., 2015) [1]. *H. pylori* was grown in a serum-free medium and at serial time points, aliquots were centrifuged and fractionated to yield culture supernatant, a soluble cellular fraction, and a membrane fraction. Samples were analyzed by single dimensional LC-MS/MS analyses and multidimensional protein identification technology (MudPIT). Here we present data showing the numbers of assigned spectra and proportional abundance of individual proteins in each of the samples analyzed, along with a calculation of the level of enrichment of individual proteins in the supernatant compared to the soluble cellular fraction.

**Specifications Table**

**Table:** 

Subject area	Biology
More specific subject area	Microbiology
Type of data	Tables
How data was acquired	Mass spectrometry using a ThermoFisher LTQ equipped with a nano-electrospray source and attached to a Nanoacuity (Waters) HPLC unit.
Data format	Filtered and analyzed
Experimental factors	Bacteria were grown in broth culture, and at serial time points, aliquots were removed, centrifuged and fractionated, to yield culture supernatant, a soluble cellular fraction, and a membrane fraction.
Experimental features	Concentrated *H. pylori* broth culture supernatants, soluble cellular fractions, and membrane preparations were analyzed by single- dimensional LC-MS/MS or multidimensional protein identification technology.
Data source location	Nashville, Tennessee, USA
Data accessibility	Data are provided in the supplementary materials accompanying this article.

## 1 Value of the data

• Comparative analyses of the proteins present in *H. pylori* broth culture supernatants, soluble bacterial fractions, and membrane fractions allow the identification of *H. pylori* proteins that are selectively released into the extracellular space.
• Analysis at multiple time points allows the identification of growth phase-dependent changes in the composition of the exoproteome.
• Further analysis of the data should allow new insights into mechanisms by which *H. pylori* proteins are released into the extracellular space.
• Further analysis of the data may allow the identification of selectively released *H. pylori* proteins that cause alterations in host cells.

## 2 Data

*H. pylori* broth culture supernatants, soluble cellular fractions, and membrane fractions were analyzed by one dimensional LC-MS/MS (1D) or multidimensional protein identification technology (MudPIT). The numbers of assigned spectra were analyzed to calculate the proportional abundance of individual proteins in samples, the enrichment of proteins in the supernatant compared to soluble bacterial fraction, and the distribution of proteins between soluble and membrane fractions (membrane localization).

Supplemental Table S1 shows all assigned spectra detected by single-dimensional LC-MS/MS analysis of supernatant and cellular fractions from five time points, collected in three independent experiments.

Supplemental Table S2 shows an analysis of merged single-dimensional LC-MS/MS data from Supplemental Table S1 to calculate enrichment of individual proteins in the supernatant compared to the soluble cellular fraction.

Supplemental Table S3 shows an analysis of merged single-dimensional LC-MS/MS data from Supplemental Table S1 for 74 putative secreted proteins (identified as described in [1]). The table shows a calculation of the enrichment of individual proteins in the culture supernatant, as well as membrane localization calculations.

Supplemental Table S4 shows all assigned spectra detected by MudPIT analysis in supernatant and cellular fractions from two time points, along with an analysis of the enrichment of individual proteins in the supernatant compared to the soluble cellular fraction.

Supplemental Table S5 shows an analysis of the MudPIT data in Supplemental Table S4 for 33 putative secreted proteins (identified as described in [1]). The table shows the enrichment of individual proteins in the supernatant compared to the soluble cellular fraction, as well as membrane localization calculations at two time points.

## 3 Experimental design, materials and methods

### 3.1 Bacterial strains and culture conditions

*H. pylori* strain 26695 was cultured at 37 °C in room air supplemented with 5% CO_2_ in a serum-free medium termed “Brucella broth filtrate”. This is a modified form of sulfite-free Brucella-cholesterol broth, prepared as described [1]. Aliquots were removed at serial time points (12, 24, 30, 36, and 48 hours post-inoculation) [1]. The samples were centrifuged at 4500 g at 4 °C for 10 min, yielding broth culture supernatants and bacterial pellets.

### 3.2 Processing of broth culture supernatants

Supernatants were passed through a 0.22 µm filter to remove any remaining bacteria, and filtered supernatants were concentrated using a 10 kDa cut-off centrifugal filter ultrafiltration unit (Amicon Ultra-15; EMD Millipore) [1]. Following buffer exchange with Tris-buffered saline (20 mM Tris, 136 mM NaCl, pH 7.4), the concentrated culture supernatants were centrifuged at 100,000 g for 2 h at 4 °C to remove outer membrane vesicles or other insoluble components [2]. The resulting supernatants were removed and further concentrated to 50 µL by ultrafiltration with a 10 kDa cut-off ultrafiltration unit.

### 3.3 Bacterial subcellular fractionation

Bacterial subcellular fractions were prepared as described previously [3] to yield a soluble cellular fraction (predicted to be enriched in cytoplasmic and periplasmic proteins) and an insoluble fraction (predicted to be enriched in membrane proteins) [1].

### 3.4 Mass spectronomic analysis of samples

Protein preparations were run about 2 cm into a 10% Bis-Tris NuPAGE gel, stained with colloidal Coomassie blue, and then subjected to in-gel trypsin digestion [4]. Samples were then analyzed by either single dimensional LC-MS/MS or multidimensional protein identification technology (MudPIT), as described in the accompanying article [1], [3]. Peptide MS/MS spectra were queried using SEQUEST (full tryptic specificity) against an *H. pylori* strain 26695 data base, to which both common contaminants and reversed versions of the proteins had been appended. Peptide identifications were filtered and collated to proteins using Scaffold 4 (Proteome Systems). Protein identifications required a minimum of 2 unique peptides per protein, and were filtered to a 5% false discovery rate (both peptide and protein).

### 3.5 Analysis of mass spectrometry data

For each sample, we calculated the proportional abundance (%abundance) of each protein detected (number of spectral counts assigned to the protein of interest divided by the total number of assigned spectral counts) [1]. To calculate the level of enrichment of an individual protein in the supernatant at a given timepoint (*E*_sup_), we compared its proportional abundance in the supernatant to its proportional abundance in the corresponding subcellular fraction containing cytoplasmic and periplasmic proteins (CP/PP) (*E*_sup_=%abundance_sup_/%abundance_CP/PP_) [1]. A value of 0.5 was added to raw data values to allow calculation of enrichment values for proteins with zero peptides detected in the CP/PP fraction. To facilitate further analysis, the levels of enrichment were expressed as log_2_ values (log_2_ *E*_sup_). To analyze the results of 1D mass spectrometric analyses, the data from three independent experiments were merged [1]. For each protein, we analyzed the statistical significance of differences in abundance of assigned spectral counts in supernatant compared to the CP/PP fraction (using Fisher׳s exact test with Bonferroni correction) [1], [3]. To investigate the subcellular localization of selectively released proteins in CP/PP and membrane fractions derived from intact bacteria, we calculated the relative abundance in the membrane fraction, using the formula [%abundance_membrane_/(%abundance_membrane_+%abundance_CP/PP_)] [1].

## Footnotes

Appendix A Supplementary data associated with this article can be found in the online version at doi:10.1016/j.dib.2015.10.008.
